# Effects of Partial Replacement of Conventional with Alternative Feeds on Nutrient Intake, Digestibility, Milk Yield and Composition of Awassi Ewes and Lambs

**DOI:** 10.3390/ani9090684

**Published:** 2019-09-15

**Authors:** Mohammad K. Aloueedat, Belal S. Obeidat, Mofleh S. Awawdeh

**Affiliations:** 1 Department of Animal Production, Faculty of Agriculture, Jordan University of Science and Technology, Irbid 22110, Jordan; mohk_89@yahoo.com; 2 Department of Veterinary Pathology and Public Health, Faculty of Veterinary Medicine, Jordan University of Science and Technology, Irbid 22110, Jordan; mawawdeh@just.edu.jo

**Keywords:** alternative feeds, Awassi ewes, digestibility, intake, milk production and composition

## Abstract

**Simple Summary:**

Alternative feeds have been used to feed animals for decades by replacing traditional feeds to increase the profit of raising livestock. In this study, alternative feeds (dried distillers grains with solubles, carob pods, olive cake, and bread by-product) were fed to Awassi ewes and ewe lambs at 0, 200, and 400 g/kg. Chemical composition was similar among diets. Feed intake, body weight change, and milk yield did not differ among diets. Cost of milk production decreased in alternative feeds containing diets, with no negative effect on health, welfare, and productivity.

**Abstract:**

Two experiments were done to assess the effects of alternative feeds (AF; dried distillers grains with solubles, carob pods, olive cake, and bread by-product) on lactating performance of ewes and digestibility and nitrogen (N) retention of lambs. Diets were: no AF (CON); 200 g/kg AF (AF200); and 400 g/kg AF (AF400). In Experiment 1, 27 Awassi ewes were randomly distributed into three groups, and each was fed one of the diets described before (9 ewes/diet). Evaluation of milk composition and yield was performed at the beginning of the experiment and on days 18, 36, and 54. In Experiment 2, 18 Awassi lambs were allocated to the same diets (6 ewe lambs/diet) during a 21-day trial (14 days housed individually in shaded pens and 7 days in metabolic cages). In Experiment 1, no differences in body weight (BW) of the ewes and their lambs were detected. With the exception of neutral detergent fiber intake, which was lower in the AF-containing diets compared with CON, dry matter, crude protein, and acid detergent fiber intake were not affected by dietary treatment. Milk yield and composition was comparable among diets, and the cost of milk production was lower in AF compared to CON diet. In Experiment 2, nutrient digestibility and N retention were not affected by the diet. Results showed the possibility of including different AF in ewe and lamb diets to mitigate production cost without negatively affecting intake, milk yield, and composition, digestibility, and animal welfare and health.

## 1. Introduction

Awassi sheep are abundant in the Mediterranean basin [1] and are raised mainly for meat, milk, and wool production. They are tolerant of semi-arid situations and resistant to diseases [2]. The large population of sheep and goat (4.3 million) in Jordan [3] and hard climatic conditions often cause a shortage in conventional feedstuffs, which limits the productivity of small ruminants and farmers’ profits in the Mediterranean basin [4]. The inclusion of agro-industrial by-products as an alternative to straw, protein sources, and cereal-based concentrates is a promising solution to this problem [5]. Due to their low cost and comparable efficiency in feeding, the usage of agro-industrial by-products in ruminant nutrition has increased, particularly in the last years [6,7].

Feeds like barley grain, soybean meal, and wheat straw are considered the major sources of nutrients for ruminants in many countries. Due to the increased international demand for these feeds, their prices have increased significantly, which has led to increased pressure on sheep producers. Researchers are therefore interested in the use of cheap alternative feeds (AF) in livestock diets, such as bread by-products (BB), carob pods (CARP) [8], olive cake (OC) [7], and dried distillers grains with solubles (DDGS) [9,10]. In addition, the use of these feeds would reduce pollution for the environment if it remains without disposal.

The first hypothesis in the experiment is that using AF in the diet of lactating Awassi ewes will not affect the performance of ewes if used properly (Experiment 1). The second hypothesis is that using AF will not negatively impact nutrient digestibility and N balance in ewe lambs (Experiment 2). The objectives of this experiment were therefore to evaluate the effects of diets including AF (DDGS, CARP, OC, and BB) on milk production and composition, BW change of lactating ewes, and growth rate of their lambs (Experiment 1), as well as on nutrient digestibility and N balance in Awassi ewe lambs (Experiment 2).

## 2. Material and Methods

### 2.1. Alternative Feeds

In the two experiments, four different alternative feeds (AF) were used: bread by-products (BB), carob pods (CARP), olive cake (OC) and dried distillers grains with solubles (DDGS). In brief, BB were obtained from the cafeteria of the restaurants at the Jordan University of Science and Technology (JUST) campus. Before mixing with the other ingredients, BB was sun-dried and ground. Carob pods (CARP) were collected from JUST campus vicinity, dried, and ground. Olive cake (OC) was obtained from local oil industry mill, transported to JUST campus and sundried, whereas DDGS was purchased locally.

### 2.2. Animals and Housing

Two experiments were conducted using the same treatment diets: no AF (CON); 200 g AF (AF200); and 400 g AF (AF400) per kg dry matter (DM). Nutrient composition of used AF is presented in Table 1. To meet lactating requirements, isonitrogenous diets (Table 2) were formulated to contain 160 g/kg crude protein (CP) [11]. Both experiments were conducted at JUST after the approval from Institutional Animal Care and Use Committee at JUST (protocol 16/03/03/521A).

### 2.3. Experiment 1

For lambing uniformity among the experimental animals, estrus was synchronized in 50 ewes using intravaginal medroxy progesterone acetate sponges (60 mg; Veramix, Pharmacia & Upjohn Co., Orangeville, Canada). Twenty-seven of the synchronized Awassi ewes (initial BW = 51.6 ± 1.86 kg; age = 3 to 4 years; second or third parity; 4–6 days in milk) were chosen for the experiment and were randomly assigned to one of the three diets (9 ewes/diet). Ewes were individually housed in shaded pens (0.75 × 1.5 m) with their single lambs. Pens were fitted with feed and water containers that allowed ad libitum access to feed and water.

Individual nutrient intake was measured during the experimental period of eight weeks (week 1 was used as an adaptation period, followed by a seven-week period for data collection) on a daily basis. Diets were offered daily at 8:00 h and orts were weighed and sampled on a daily basis. The animals BW was measured on days 0, 28, and 56 of the study. On days 18, 36, and 54, milk yield was recorded at 8:00 h by hand milking. Milk yield was calculated over a 24 h period, and lambs were separated from their dams 12 h before milking.

### 2.4. Experiment 2

Eighteen female lambs (BW = 38.1 ± 1.0 kg; age = 7 to 8 months) were randomly assigned to one of the three diets (6 ewe lambs/diet) to measure N balance and digestibility. The experiment lasted for 21 days [adaptation period; 14 days housed in individual pens (0.75 × 1.5 m) and collection period; 7 days in metabolic cages (0.8 × 1.05)]. Lambs were housed, fed, and controlled under the same regime used in Experiment 1. They were placed in metabolic cages for fecal and urinary collection from day 15 to day 21. Daily fecal output was collected, weighed, and 10 g/100 g was kept for subsequent analyses. Urine was collected using plastic containers, weighed, and 5 g/100 g was kept (at −20 °C) to evaluate N balance. Each container contained 50 mL of 6 N HCL to prevent losses of ammonia.

### 2.5. Laboratory Procedures 

In Experiment 1, a 125 mL milk sample was collected from each ewe on the assigned days mentioned previously and analyzed for total solids (in a forced oven at 55 °C), fat (Gerber method, Gerber Instruments, K. Schnider and Co. AG; 8307 Langhag, Effretikon), and CP (Kjeldahl procedure; N × 6.38). For chemical analysis, diet and feed refusal samples were dried in a forced air oven at 105 °C overnight to determine final DM and crude protein content (CP) [12]. Neutral detergent fiber (NDF) and acid detergent fiber (ADF) were analyzed using an ANKOM^2000^ fiber analyzer (ANKOM Technology Corp., Fairport, NY, USA).

At the end of the experiment, samples of feces and urine were composited for each lamb. Fecal samples were dried in a forced air oven at 55 °C for 4 days. Fecal and dietary samples were then ground to pass through a 1 mm screen and were stored in plastic bags at room temperature for subsequent analyses using the procedures described by the AOAC [12]. Diet and fecal samples were analyzed for DM, CP, NDF, and ADF, as described previously. 

For determination of N balance, samples of feces and urine were composited for each lamb and analyzed for N content to calculate N intake and N lost in feces and urine to measure N retention as described by Obeidat et al. [13].

### 2.6. Statistical Analyses

Using the mixed procedure of SAS (version 8.1, 2000, SAS Inst. Inc., Cary, NC, USA), data were analyzed as a completely randomized design. In Experiment 1, milk production and ewe and lamb BW were analyzed using repeated measures ANOVA that included treatment, day, and treatment × day interaction. As no treatment × day interaction was detected, only the main effects are presented. In Experiment 2, the treatment was the fixed effect, and the animal was a random effect. Least square means of the MIXED procedures of SAS were used to detect significant differences among means. In both experiments, a probability of *p* < 0.05 was considered as significant.

## 3. Results

### 3.1. Nutrients Intake, Body Weight, Milk Production, and Composition (Experiment 1)

Intake of DM, CP, and ADF did not differ (*p* ≥ 0.25) between treatment groups throughout the study. Intake of NDF was greater (*p* < 0.01) in the animal fed the CON diet compared to the AF200 and AF400 diets. Initial BW, final BW, and BW change of ewes did not differ (*p* ≥ 0.70) among dietary treatments (Table. 3). Lamb weaning BW, total gain, and pre-weaning average daily gain were not different (*p* ≥ 0.52) across dietary treatments (Table 3). No differences (*p* ≥ 0.46) were detected in milk yield, composition (fat, protein, and total solids), or yield of any analyzed fraction. Feed intake-to-milk yield ratio was similar (*p* = 0.97) across treatments (Table 4).

### 3.2. Economic Evaluation 

Inclusion of 200 and 400 g/kg of AF in the diet decreased diets cost by 10 and 20%, respectively, compared with the CON diet (Table 2). Milk cost was the lowest (*p* ≤ 0.05) in the AF400 diet, followed by the AF200 (*p* ≤ 0.05) and CON diets.

### 3.3. Digestibility and N Balance (Experiment 2)

Dry matter, CP, NDF, and ADF intake of lambs are shown in Table 5. No differences (*p* ≥ 0.18) were observed in nutrient intake among treatment groups throughout the study. Nutrient digestibility and N balance results are presented in Table 6. No differences (*p* ≥ 0.21) were observed in nutrient digestibility among treatments. Similarly, no differences (*p* ≥ 0.15) among groups were observed in N intake, N lost in feces, N lost in the urine, N retained (g/d), or N retention (g/100g).

## 4. Discussion

### 4.1. Nutrient Intake, Body Weight, Milk Production, and Composition

All animals completed the experiment without any noticeable health problems or disorders. In agreement with our results, previous studies have shown that the use of AF did not affect the health status of the animals [6,7,8,9,10]. In the current study, no effect on DM, CP, and ADF intake was found. The increased intake of NDF for the CON diet can be attributed to the higher NDF content of this diet compared to the AF200 and AF400 diets, because of the higher level of NDF from wheat straw in the CON group. Consistent with our study, OC [14], CARP [15], and BB [16] had no effect on the intake of DM, OM, CP, NDF, and ADF in Awassi sheep in previous studies. Consistently, Kleinschmit et al. [17] and Schingoethe et al. [18] reported that DM intake was not reduced by including DDGS in the diet until the incorporation rate exceeded 200 to 300 g/kg of dietary DM. Moreover, Felix et al. [19] reported that increased dietary DDGS diets (0, 200, 400, and 600 g/kg DM) did not affect DM intake in feedlot lambs.

In contrast, Hindiyeh et al. [20] found that different levels of BB (100, 200, and 300 g/kg DM) decreased DM, OM, CP, NDF, and metabolizable energy (ME) intake in a fattening diet for Awassi lamb compared to a control diet. Hence, the different response to the BB intake could be attributed to the components of this by-product, animal species, basal diet composition, and inclusion level [16]. The similarity in nutrient intake (apart from NDF intake) in all groups in our study could be attributed to the fact that the inclusion of AF at 200 or 400 g/kg DM did not change the nutrient composition of all diets. Our experiment feed intake results are in agreement with previous studies indicating that these AF are palatable and easy to be consumed by the animals [14,15,16,17,18,20].

Ewes’ BW changes and the lambs’ average daily gain (ADG) did not differ between treatments. Similarly, Belibasakis [21] and Omar et al. [22] reported no negative effect on the ADG and BW of lambs with the inclusion of 150 to 250 g/kg DM of OC. Obeidat et al. [15] also found that different levels of CARP inclusion (0, 150, and 250 g/kg DM) did not affect BW change or ADG. Moreover, BW change and ADG of nursing Awassi ewes and their lambs did not differ between treatment diets with 100, 150, and 200 g/kg DM of BB [16]. Alshdaifat and Obeidat [9] reported no impact on BW change of nursing ewes or ADG of their suckling lambs by replacing soybean meal with DDGS. Morricall [23] also found no difference in the BW of ewes and lamb total gain when nursing ewes were fed DDGS diets compared to basal diets.

Similar to our results, Obeidat et al. [8]; CARP), Obeidat et al. [16] (BB), and Janicek et al. [24] (DDGS) observed no effects on milk production and composition when AF were included in the diet. Recently, Keles et al. [25] supplemented dairy Saanen goats with OC silage at 0, 100, and 200 g/kg of dietary DM and observed no effect on milk yield, CP, lactose, energy corrected milk (ECM), fat corrected milk (FCM), or feed efficiency, while fat content and fat production increased. Diets containing OC silage may have produced milk with a higher fat content due to its high level of fiber (NDF and ADF) and, subsequently, more volatile fatty acids were generated (mainly acetate; the major precursor for fatty acid synthesis).

Moreover, Hassan et al. [26] reported that lactating Zaraibi goats fed diets containing CARP at levels of 25 and 50 g/kg DM had greater milk production and protein and fat percentages than when supplemented with 100 g/kg of CARP. This could be linked to an increase in the metabolizable protein supply associated with condensed tannins (CT). Protein-CT complex is preserved from microbial degradation and, subsequently, digested and absorbed as amino acids in the small intestine [27].

Kleinschmit et al. [28] found that DDGS contains an average 200–300 g CP/kg, of which about 50–55% is rumen undegraded protein (RUP). Consequently, Kleinchmit et al. [17] and Pamp et al. [29] reported that milk yield increased with the inclusion of DDGS in diets. Leonardi et al. [30] also reported a linear increase in milk yield and milk protein yield with an increasing dietary DDGS (at 0, 50, 100, and 150 g/kg of dietary DM) in dairy cows. Owen and Larson [31] found that milk production in early lactating cows increased when DDGS was included at 188 g/kg DM but decreased when DDGS was included at 358 g/kg of the diet DM. These findings may be due to lower protein absorbability and lysine deficiencies.

### 4.2. Economic Evaluation

Milk cost was lower for AF diets than for the control diet. In an earlier study, Obeidat et al. [16] reported that the milk production cost decreased for ewes fed BB at levels of 150 and 200 g/kg of dietary DM. The decrease in the cost of milk production is closely related to diet cost and is considered beneficial for milk producers, while not affecting health, welfare, and efficiency. Similar results to those of Obeidat et al. [8] were reported with partially replacing of barley grains with CARP. If AF negatively affects intake, digestibility, and diet utilization, the cost benefits of the AF should be considered together with overall production aspects. In our study, there were no negative effects on any of these parameters, and consequently, the use of the studied AF should be taken into consideration by ewe-milk producers to reduce their operational (i.e., feeding) costs and the cost of milk production and maximize their operating profits.

### 4.3. Intake, Digestibility, and N Balance

Similarly to our results (1 and 2), Tufarelli et al. [32] did not find any differences in intake of DM and CP when lambs were fed diets with different levels of partly destoned exhausted OC (0, 100, or 200 g/kg DM). Furthermore, Silanikove et al. [33] observed no change in DM intake when feeding 520 g/kg DM CARP to Anglo-Nubian goats, and Benchaar et al. [34] reported similar DM intake when supplementing dairy cows with 150 g CT/kg. Reed [35] and Silanikove et al. [36], however, reported that feed intake decreased due to the high levels of CT present in the CARP reducing palatability. In contrast to these finding, Afzalzadeh et al. [37] reported that diets containing BB at different levels (0, 60, 120, and 250 g/kg of dietary DM) had no effect on DM intake in fattening Zandi lambs. Similarly, Felix et al. [19] reported that increased dietary DDGS (0, 200, 400, and 600 g/kg DM) did not affect DM intake in feedlot lambs, and McKeown et al. [38] reported that DDGS from different sources of cereals had no effect on ADG of feedlot lambs.

The nutrient digestibility results were consistent with those of Awawdeh and Obeidat [14], who reported no difference in the digestibility of DM, OM, CP, and NDF with the dietary inclusion of OC. Tufarelli et al. [32], however, reported that DM and fiber (NDF and ADF) digestibility diminished in diets containing partly destoned exhausted OC (100 or 200 g/kg of dietary DM) compared to a control diet. This finding could be explained by Feggeros and Kalaisakes [39], who reported that digestibility of OC was very low because of the greater levels of lignin and tannin. Moreover, Filya et al. [40] observed that poor digestibility values are a consequence of the close association of proteins with lignocellulosic compounds.

In contrast to these findings, Awassi lambs that were fed a diet containing 125 and 250 g/kg DM of CARP showed no negative effects on nutrient digestibility [15]. Similarly, Benchaar et al. [34] found that a diet supplemented with 150 g/kg CT produced no differences in apparent DM, OM, CP, NDF, and ADF digestibility. Silanikove et al. [33] and Obeidat et al. [8], however, found that CARP had an adverse effect on DM digestibility and the digestibility of other nutrients compared to a control diet. These effects could be attributed to the high CT levels in CARP, which can diminish proteolysis rate, restraining the development of rumen proteolytic microorganisms and reducing the digestibility of nutrients, especially CP. Provenza [41] showed that a high level of tannins in animal feed could potentially reduce the nutritional value as tannins bind to proteins and reduce their availability to the ruminal microflora. Obeidat et al. [16], however, reported that DM, OM, CP, NDF, and ADF digestibility was similar across treatments with partial replacement of barley with BB at 0, 100, 150, and 200 g/kg of dietary DM in nursing Awassi ewes diets. Moreover, Felix et al. [19] found that diets containing DDGS (0, 200, 400, and 600 of dietary DM) did not affect ether extract (EE), NDF, and ADF digestibility of lambs.

In contrast, Winterholler et al. [42] reported that when spring-calving beef cows were fed DDGS at different levels (0.77, 1.54, and 2.31 kg/d), the digestibility of NDF, ADF, CP, and fat increased. This result could be explained by Lemenager et al. [43], who found that DDGS led to the high digestibility of nutrients, including NDF and ADF. The use of isonitrogenous and isocaloric diets in our study may have prevented the negative effects on production parameters that were previously observed in other studies, but further studies are needed in this area to validate these findings.

Our N balance results are in line with Awawdeh and Obeidat [14], who found that diets containing OC did not affect the N intake or the N excreted in the feces and urine of Awassi lambs. Consequently, retained N was not different among treatments. This is similar to Almira et al. [44], who found that N intake was not affected by the ratio of BB to cornmeal and observed no influence of BB on the excretion of urinary and fecal N or on N balance. In contrast, Felix et al. [19] reported that increasing DDGS levels in lamb diets increased N intake, N digestibility, and urinary N output, while N retention decreased. In addition, Benchaar et al. [45] observed that N intake, fecal N, urinary N, total N excretion, and retained N increased with increasing DDGS (0, 100, 200, and 300 g/kg DM) in dairy cows. Gurung et al. [46] also found that when goats were fed diets with increasing DDGS levels (0, 127, 254, 381 g/kg DM), urinary N increased because DDGS is a good source of RUP, while fecal N, urinary N, retention N, and retained N were similar across treatments.

## 5. Conclusions

Results of the current study indicate that inclusion of AF in the diet of lactating ewes did not affect health, welfare, global production efficiency, feed intake, milk production and composition, nutrient digestibility, and N balance in lambs, but did reduce diet and milk production cost.

## Figures and Tables

**Table 1:** Chemical composition of alternative feeds (AF) * used in the experiment.

Nutrient **	CARP	DDGS	OC	BB
DM, g/kg	915	910	893	915
CP, g/kg DM	82	273	93	124
NDF, g/kg DM	213	272	530	24
ADF, g/kg DM	150	102	348	6.1
ME, Mcal/kg ***	2.87	2.90	1.03	3.30

* Alternative feeds (by-products, BB; carob pods, CARP; olive cake, OC; dried distillers grains with solubles, DDGS. ** Nutrient (dry matter, DM; crude protein, CP; neutral detergent fiber, NDF; acid detergent fiber, ADF; metabolizable energy, ME. *** Calculated based on tabular values (NRC, 2007).

**Table 2:** Ingredients, cost and chemical composition of diets containing alternative feeds (AF) for lactating Awassi ewes and lambs.

Item	Diets *		
	CON	AF200	AF400
**Ingredients (g/kg DM)**			
Barley grain	555	475	327
Soybean meal, 440 g/kg CP (solvent)	165	125	103
Carob pods	0	40	80
Dried distillers grains with solubles	0	70	140
Olive cake	0	50	100
Bread by-products	0	40	80
Wheat straw	260	180	150
Salt	10	10	10
Limestone	9	9	9
Mineral vitamin premix **	1	1	1
Cost ($/1000 kg)	359	319	287
**Chemical composition**			
Dry matter, g/kg	915	913	911
Crude protein, g/kg DM	160	158	157
Neutral detergent fiber, g/kg DM	320	279	259
Acid detergent fiber, g/kg DM	137	124	127
Metabolizable energy, Mcal/kg ***	2.3	2.4	2.4

* Diets were: no AF (CON); 200 AF (AF200) of DM; and 400 g/kg AF (AF400) of dietary DM. ** Composition per kg contained: Vitamin A, 600,000 IU; vitamin D3, 200,000 IU; vitamin E, 75 mg; vitamin K3, 200 mg; vitamin B1, 100 mg; vitamin B5, 500 mg; lysine 0.5%; DL-methionine, 0.15%; manganese oxide, 4000 mg; ferrous sulphate, 15,000 mg; zinc oxide, 7000 mg; magnesium oxide, 4000 mg; potassium iodide, 80 mg; sodium selenite, 150 mg; copper sulphate, 100 mg; cobalt phosphate, 50 mg; dicalcium phosphate, 10,000 mg. *** Calculated based on tabular values (NRC, 2007).

**Table 3:** Intake and BW change in lactating Awassi ewes fed diets containing alternative feeds (AF).

Item	Diets *			SEM	*p*-Value
	CON	AF200	AF400		
**Ewe**					
Intake, g/d					
Dry matter	2502	2547	2625	102.6	0.60
Crude protein	400	402	412	16.3	0.81
Neutral detergent fiber	800 ^b^	709 ^a^	680 ^a^	29.5	0.001
Acid detergent fiber	342	315	332	13.3	0.25
Initial BW, kg	50.6	52.9	51.2	1.86	0.49
Final BW, kg	58.0	59.4	58.6	2.34	0.80
BW change, kg	7.4	6.5	7.5	1.14	0.69
**Lambs**					
Initial BW, kg	5.5	5.4	5.8	0.41	0.79
Final BW, kg	18.7	19.8	19.1	0.97	0.70
Total gain, kg	13.2	14.4	13.3	0.79	0.52
ADG, g	235	257	238	14.2	0.52

* Diets were no AF (CON); 200 AF (AF200) of dietary DM; or 400 g/kg AF (AF400) of dietary DM. ^ab^ Within a row means without a common superscript differ (*p* < 0.05).

**Table 4:** Milk yield and composition in lactating Awassi ewes fed diets containing alternative feeds (AF).

Item	Diets *			SEM	*p*-Value
	CON	AF200	AF400		
Milk Yield, g/d	950	929	893	101.5	0.92
Milk composition, g/kg					
Fat	48.8	49.4	50.0	0.65	0.45
Protein	63.4	63.6	63.0	1.87	0.96
Total solids	153	156	157	4.01	0.81
Milk Yield, g/d					
Fat	46.8	46.1	44.5	5.36	0.95
Protein	60.4	59.6	56.2	7.0	0.90
Total solids	145.2	146.0	141.0	17.67	0.97
Feed to milk	3.07	2.95	3.01	0.314	0.96
Cost of milk (US$/kg milk)	1.11 ^a^	0.94 ^b^	0.86 ^c^	0.022	≤0.05

* Diets were no AF (CON); 200 AF (AF200) of dietary DM; or 400 g/kg AF (AF400) of dietary DM. ^abc^ Within a row means without a common superscript differ (*p* < 0.05).

**Table 5:** Intake of nutrients in Awassi lambs fed diets containing alternative feeds (AF).

Item	Diets *			SEM	*p*-Value
	CON	AF200	AF400		
**Intake**					
Dry matter, g/d	1185	1297	1205	75.2	0.55
Crude protein, g/d	206	224	212	13.1	0.62
Neutral detergent fiber, g/d	375	401	370	23.3	0.60
Acid detergent fiber, g/d	162	161	149	10.0	0.18

* Diets were: no AF (CON); 200 AF (AF200) of dietary DM; or 400 AF (AF400) of dietary DM.

**Table 6:** Nutrient digestibility and N balance in Awassi lambs fed diets containing alternative feeds (AF).

Item	Diets *			SEM	*p*-Value
	CON	AF200	AF400		
**Digestion coefficients**					
Dry matter	0.77	0.78	0.73	0.019	0.21
Crude protein	0.78	0.78	0.75	0.020	0.56
Neutral detergent fiber	0.57	0.59	0.54	0.044	0.71
Acid detergent fiber	0.48	0.51	0.48	0.042	0.86
**N Balance**					
N intake, g/d	32.9	35.8	33.9	2.10	0.62
N feces, g/d	7.3	8.0	8.5	0.87	0.61
N urine, g/d	12.2	10.4	9.6	0.98	0.15
N retained, g/d	13.5	17.4	15.9	1.78	0.31
N retention, g/100 g	40.5	48.5	46.2	3.51	0.29

* Diets were: no AF (CON); 200 AF (AF200) of dietary DM; or 400 AF (AF400) of dietary DM.
